# Supplementary material for: Studies on geochemical characteristics and biomineralization of Cambrian phosphorites, Zhijin, Guizhou Province, China
Source: PLoS One. 2023 Feb 10;18(2):e0281671. doi: 10.1371/journal.pone.0281671 (PMC9916593; doi:10.1371/journal.pone.0281671)
Supplement: S1 Table — (DOCX) [file pone.0281671.s001.docx]

**S1 Table** Concentrations of major elements (wt%) of the Zhijin phosphorites samples in the lower Cambrian strata.

| Sample | ZLX1-1 | ZLX2-5 | ZLX3-2 | ZGH-4-1 | ZGZW-1-1 | ZGZW-5-1 | ZGZW-8-2 | ZDMC-2-1 | ZDMC-3-1 | ZYCG-1 | ZYCG-5 | ZYCG-8 |
| --- | --- | --- | --- | --- | --- | --- | --- | --- | --- | --- | --- | --- |
| Al_2_O_3_ | 0.72 | 3.07 | 2.78 | 3.29 | 1.59 | 0.53 | 1.31 | 1.34 | 0.27 | 0.67 | 0.26 | 1.25 |
| BaO | 0.13 | 0.06 | 0.04 | 0.27 | 0.04 | 0.03 | 0.06 | 0.05 | 0.46 | 0.03 | 0.04 | 0.02 |
| CaO | 37.1 | 35.8 | 24.7 | 22.6 | 33.7 | 31.6 | 49.5 | 23.9 | 28.5 | 48 | 46.8 | 22.3 |
| Fe_2_O_3_ | 0.63 | 1.71 | 2.11 | 4.78 | 0.89 | 0.59 | 1.81 | 0.94 | 0.30 | 0.94 | 0.62 | 2.19 |
| K_2_O | 0.24 | 1.08 | 0.90 | 1.04 | 0.41 | 0.17 | 0.35 | 0.54 | 0.08 | 0.22 | 0.09 | 0.42 |
| MgO | 9.92 | 3.33 | 1.92 | 0.65 | 0.72 | 8.73 | 0.39 | 13.65 | 20.1 | 5.18 | 6.85 | 8.86 |
| MnO_2_ | 0.08 | 0.10 | 0.06 | 0.01 | 0.02 | 0.16 | 0.23 | 0.14 | 0.10 | 0.06 | 0.06 | 0.11 |
| Na_2_O | 0.04 | 0.06 | 0.05 | 0.05 | 0.06 | 0.03 | 0.06 | 0.01 | 0.01 | 0.08 | 0.08 | 0.02 |
| P_2_O_5_ | 16.59 | 22.45 | 16.25 | 19.55 | 23.8 | 13.91 | 35.7 | 2.91 | 0.22 | 28.7 | 26.6 | 7.45 |
| SiO_2_ | 10.70 | 22.50 | 42.80 | 43.10 | 33.2 | 22.80 | 6.55 | 24.10 | 5.06 | 2.25 | 1.16 | 36.40 |
| SO_3_ | 0.13 | 0.18 | 1.16 | 1.36 | 0.21 | 0.32 | 0.19 | 0.54 | 0.29 | 0.18 | 0.14 | 0.07 |
| SrO | 0.06 | 0.09 | 0.06 | 0.03 | 0.08 | 0.05 | 0.12 | 0.01 | 0.01 | 0.09 | 0.08 | 0.03 |
| TiO_2_ | 0.03 | 0.15 | 0.13 | 0.19 | 0.06 | 0.03 | 0.07 | 0.05 | 0.01 | 0.05 | 0.04 | 0.04 |
