# Supplementary material for: Studies on geochemical characteristics and biomineralization of Cambrian phosphorites, Zhijin, Guizhou Province, China
Source: PLoS One. 2023 Feb 10;18(2):e0281671. doi: 10.1371/journal.pone.0281671 (PMC9916593; doi:10.1371/journal.pone.0281671)
Supplement: S2 Table — (DOCX) [file pone.0281671.s002.docx]

**S2 Table** Concentrations of trace and rare earth elements (ppm) of the Zhijin phosphorites samples in the lower Cambrian strata.

| Sample | ZDMC-1-1 | ZDMC-2-1 | ZDMC-2-2 | ZDMC-3-1 | ZGZW-1-1 | ZGZW-1-2 | ZGZW-3-2 | ZGZW-3-3 | ZGZW-4-2 | ZGZW-5-1 | ZGZW-6-1 | ZGZW-6-3 | ZGZW-7-1 | ZGZW-8-2 |
| --- | --- | --- | --- | --- | --- | --- | --- | --- | --- | --- | --- | --- | --- | --- |
| La(ppm) | 79.30 | 40.60 | 82.10 | 5.19 | 72.60 | 62.40 | 86.50 | 163.00 | 130.00 | 111.00 | 149.00 | 158.00 | 80.60 | 110.00 |
| Ce | 59.70 | 34.10 | 69.90 | 4.61 | 134.00 | 54.80 | 155.00 | 124.00 | 95.40 | 86.30 | 126.00 | 133.00 | 160.00 | 89.10 |
| Pr | 13.70 | 7.22 | 48.60 | 0.82 | 37.60 | 11.00 | 44.50 | 33.40 | 25.30 | 22.30 | 34.90 | 37.10 | 46.60 | 62.20 |
| Nd | 56.90 | 30.20 | 208.00 | 3.43 | 154.00 | 43.20 | 185.00 | 141.00 | 103.00 | 95.20 | 154.00 | 164.00 | 208.00 | 278.00 |
| Sm | 11.00 | 5.84 | 42.50 | 0.77 | 30.50 | 7.96 | 36.30 | 27.10 | 19.80 | 18.20 | 31.90 | 34.00 | 42.50 | 61.10 |
| Eu | 2.31 | 1.49 | 10.51 | 0.72 | 7.16 | 1.88 | 8.69 | 6.05 | 4.80 | 4.70 | 10.87 | 10.56 | 13.25 | 17.91 |
| Gd | 11.92 | 7.00 | 45.02 | 0.98 | 33.80 | 8.46 | 40.86 | 29.42 | 21.84 | 20.64 | 34.89 | 35.64 | 45.38 | 62.90 |
| Tb | 1.85 | 0.99 | 6.39 | 0.11 | 4.73 | 1.26 | 5.53 | 4.38 | 3.28 | 2.98 | 4.43 | 4.82 | 5.95 | 8.29 |
| Dy | 11.20 | 6.16 | 38.70 | 0.66 | 29.50 | 7.68 | 34.90 | 27.50 | 19.90 | 18.20 | 25.90 | 27.90 | 35.70 | 48.30 |
| Ho | 2.71 | 1.48 | 8.40 | 0.16 | 6.58 | 1.79 | 7.97 | 6.19 | 4.49 | 4.11 | 5.75 | 6.13 | 7.85 | 10.40 |
| Er | 7.33 | 4.15 | 21.90 | 0.36 | 17.50 | 4.83 | 20.30 | 16.20 | 12.00 | 10.40 | 14.40 | 15.50 | 19.60 | 26.40 |
| Tm | 0.91 | 0.55 | 2.66 | 0.07 | 2.07 | 0.64 | 2.45 | 1.90 | 1.45 | 1.24 | 1.68 | 1.71 | 2.27 | 3.17 |
| Yb | 4.68 | 2.84 | 12.50 | 0.31 | 9.86 | 3.48 | 11.90 | 9.30 | 6.97 | 5.59 | 7.60 | 7.88 | 10.00 | 15.10 |
| Lu | 0.69 | 0.45 | 1.68 | 0.05 | 1.28 | 0.45 | 1.56 | 1.22 | 0.97 | 0.77 | 1.01 | 1.03 | 1.27 | 1.99 |
| Y | 110.00 | 62.60 | 107.00 | 6.24 | 96.50 | 78.30 | 115.00 | 87.60 | 166.00 | 146.00 | 79.50 | 88.00 | 110.00 | 148.00 |
| V | 11.00 | 11.60 | 18.10 | 24.50 | 25.40 | 127.00 | 58.20 | 33.40 | 34.40 | 24.80 | 61.20 | 40.10 | 95.30 | 44.50 |
| Cr | 7.90 | 6.58 | 15.40 | 20.90 | 11.40 | 51.70 | 21.90 | 8.89 | 16.30 | 4.95 | 6.78 | 9.90 | 17.60 | 12.00 |
| Co | 20.60 | 9.58 | 10.30 | 2.69 | 12.60 | 10.70 | 14.70 | 8.49 | 17.40 | 7.08 | 5.44 | 7.89 | 8.47 | 11.30 |
| Ni | 18.20 | 11.99 | 15.87 | 15.44 | 64.16 | 101.75 | 64.93 | 119.00 | 150.04 | 15.69 | 13.88 | 19.66 | 24.40 | 42.60 |
| Mo | 1.43 | 0.65 | 0.73 | 0.42 | 20.30 | 3.61 | 16.60 | 4.37 | 16.50 | 1.80 | 0.79 | 1.06 | 1.32 | 5.57 |
| Ag | 0.75 | 0.87 | 2.67 | 0.54 | 2.09 | 1.05 | 3.92 | 1.58 | 1.92 | 1.57 | 3.26 | 3.66 | 2.80 | 2.04 |
| As | 11.50 | 10.80 | 13.10 | 8.81 | 11.10 | 11.50 | 12.20 | 9.55 | 14.40 | 8.77 | 12.40 | 9.60 | 13.10 | 11.80 |
| Sb | 4.59 | 4.36 | 6.99 | 5.83 | 18.46 | 52.43 | 4.85 | 8.07 | 12.31 | 2.91 | 23.55 | 4.31 | 7.29 | 3.45 |
|  |  |  |  |  |  |  |  |  |  |  |  |  |  |  |
| Sample | ZLX1-1 | ZLX1-3 | ZLX1-4 | ZLX2-1 | ZLX2-2 | ZLX2-5 | ZLX2-8 | ZLX3-1 | ZLX3-2 | ZGH-1 | ZGH-4-1 | ZGH-4-2 | ZGH-5-2 |  |
| La(ppm) | 128.00 | 118.00 | 134.00 | 103.00 | 151.00 | 95.20 | 109.00 | 77.00 | 132.00 | 17.90 | 111.00 | 14.50 | 35.80 |  |
| Ce | 86.60 | 160.00 | 97.20 | 69.10 | 121.00 | 163.00 | 91.60 | 138.00 | 115.00 | 17.60 | 131.00 | 17.90 | 62.70 |  |
| Pr | 23.80 | 49.60 | 28.20 | 18.80 | 34.20 | 43.90 | 56.30 | 36.60 | 30.20 | 3.14 | 31.70 | 3.19 | 6.96 |  |
| Nd | 99.30 | 211.00 | 119.00 | 77.00 | 146.00 | 189.00 | 244.00 | 159.00 | 132.00 | 12.90 | 133.00 | 13.70 | 21.70 |  |
| Sm | 18.00 | 39.70 | 21.90 | 14.00 | 27.10 | 35.70 | 49.40 | 32.00 | 26.20 | 2.77 | 30.20 | 4.11 | 3.22 |  |
| Eu | 4.46 | 9.46 | 5.81 | 3.38 | 5.93 | 10.93 | 12.11 | 8.66 | 9.30 | 0.67 | 6.62 | 2.59 | 0.57 |  |
| Gd | 21.10 | 45.76 | 26.33 | 16.13 | 30.53 | 43.49 | 55.84 | 37.60 | 31.39 | 2.76 | 30.19 | 3.90 | 2.64 |  |
| Tb | 2.85 | 6.42 | 3.55 | 2.26 | 4.28 | 5.96 | 7.62 | 5.05 | 4.16 | 0.36 | 4.42 | 0.58 | 0.41 |  |
| Dy | 18.30 | 40.00 | 21.70 | 14.50 | 26.20 | 37.10 | 46.80 | 31.40 | 25.30 | 2.21 | 25.50 | 3.52 | 2.64 |  |
| Ho | 4.18 | 9.13 | 5.01 | 3.27 | 5.96 | 8.54 | 10.40 | 7.02 | 5.84 | 0.51 | 5.21 | 0.69 | 0.67 |  |
| Er | 11.20 | 23.40 | 12.70 | 8.55 | 15.40 | 21.60 | 26.10 | 18.10 | 14.70 | 1.33 | 12.90 | 2.01 | 1.98 |  |
| Tm | 1.36 | 2.77 | 1.55 | 1.09 | 1.77 | 2.45 | 3.04 | 2.15 | 1.71 | 0.17 | 1.61 | 0.37 | 0.32 |  |
| Yb | 6.34 | 13.20 | 7.09 | 5.18 | 8.28 | 10.80 | 14.00 | 9.97 | 7.68 | 0.89 | 7.83 | 2.58 | 2.27 |  |
| Lu | 0.89 | 1.67 | 0.91 | 0.65 | 1.10 | 1.41 | 1.80 | 1.27 | 1.01 | 0.13 | 1.01 | 0.36 | 0.34 |  |
| Y | 159.00 | 152.00 | 85.80 | 137.00 | 98.50 | 133.00 | 151.00 | 112.00 | 91.40 | 23.50 | 62.90 | 23.60 | 17.10 |  |
| V | 12.30 | 15.20 | 10.60 | 12.10 | 73.70 | 54.20 | 18.20 | 22.00 | 39.00 | 63.10 | 84.70 | 139.00 | 178.00 |  |
| Cr | 34.80 | 73.90 | 22.30 | 10.70 | 48.50 | 24.00 | 14.60 | 18.80 | 14.00 | 13.70 | 44.00 | 426.00 | 77.10 |  |
| Co | 8.10 | 7.36 | 15.60 | 7.37 | 8.42 | 8.76 | 10.80 | 22.60 | 32.20 | 18.10 | 10.70 | 90.20 | 11.30 |  |
| Ni | 112.96 | 230.24 | 77.70 | 36.22 | 32.25 | 25.61 | 128.49 | 28.97 | 18.02 | 19.14 | 38.11 | 3.13 | 8.17 |  |
| Mo | 29.30 | 63.70 | 18.80 | 6.32 | 5.85 | 2.54 | 7.07 | 11.50 | 1.85 | 20.90 | 11.00 | 14.60 | 38.50 |  |
| Ag | 1.00 | 2.10 | 3.12 | 1.18 | 3.35 | 2.04 | 27.50 | 3.56 | 4.02 | 0.51 | 5.13 | 9.50 | 0.92 |  |
| As | 9.17 | 9.55 | 8.43 | 12.70 | 8.48 | 10.20 | 17.80 | 15.30 | 15.80 | 10.60 | 29.80 | 17.70 | 12.40 |  |
| Sb | 2.45 | 1.45 | 78.60 | 3.32 | 4.68 | 8.53 | 18.87 | 12.47 | 9.85 | 3.81 | 18.87 | 10.50 | 5.55 |  |
|  |  |  |  |  |  |  |  |  |  |  |  |  |  |  |
| Sample | ZX-2 | ZX-4-1 | ZX-5 | ZX-7 | ZYCG-1 | ZYCG-2 | ZYCG-3 | ZYCG-4 | ZYCG-5 | ZYCG-6 | ZYCG-7 | ZYCG-8 | ZYCG-9 | ZYCG-10 |
| La(ppm) | 2.70 | 97.00 | 131.00 | 0.38 | 65.00 | 85.60 | 95.60 | 98.70 | 76.20 | 102.00 | 112.00 | 80.90 | 99.10 | 80.00 |
| Ce | 3.19 | 87.10 | 112.00 | 0.71 | 130.00 | 63.50 | 180.00 | 182.00 | 133.00 | 163.00 | 70.40 | 67.60 | 90.10 | 71.30 |
| Pr | 0.67 | 18.10 | 26.30 | 0.09 | 34.90 | 47.80 | 53.20 | 53.90 | 40.00 | 53.20 | 57.30 | 15.90 | 60.80 | 17.30 |
| Nd | 3.02 | 73.40 | 109.00 | 0.31 | 147.00 | 204.00 | 230.00 | 233.00 | 169.00 | 226.00 | 241.00 | 68.40 | 277.00 | 76.10 |
| Sm | 0.78 | 14.70 | 23.10 | 0.08 | 27.40 | 41.30 | 43.70 | 44.70 | 31.40 | 41.40 | 47.00 | 13.60 | 60.80 | 15.10 |
| Eu | 0.22 | 3.98 | 5.84 | 0.02 | 7.61 | 10.40 | 11.12 | 11.19 | 6.89 | 9.29 | 10.27 | 3.89 | 17.76 | 4.88 |
| Gd | 0.76 | 16.67 | 26.78 | 0.08 | 31.57 | 45.06 | 47.68 | 49.18 | 34.02 | 46.76 | 49.78 | 14.20 | 62.27 | 16.42 |
| Tb | 0.11 | 2.37 | 3.68 | 0.01 | 4.46 | 6.19 | 6.88 | 7.31 | 5.11 | 6.81 | 7.67 | 2.04 | 8.86 | 2.28 |
| Dy | 0.57 | 14.10 | 22.60 | 0.05 | 26.80 | 37.40 | 41.90 | 44.30 | 32.00 | 43.40 | 47.80 | 12.20 | 50.10 | 13.60 |
| Ho | 0.12 | 3.21 | 5.07 | 0.01 | 6.27 | 8.59 | 9.66 | 10.00 | 7.58 | 10.00 | 11.10 | 2.79 | 11.00 | 2.92 |
| Er | 0.30 | 8.32 | 13.00 | 0.04 | 16.40 | 22.20 | 25.00 | 25.70 | 19.00 | 25.60 | 27.40 | 7.44 | 27.30 | 7.40 |
| Tm | 0.05 | 1.04 | 1.55 | 0.01 | 2.02 | 2.72 | 2.98 | 3.16 | 2.33 | 3.06 | 3.40 | 0.89 | 3.27 | 0.87 |
| Yb | 0.20 | 4.61 | 7.54 | 0.03 | 9.59 | 12.60 | 14.20 | 14.20 | 10.90 | 14.10 | 15.70 | 4.00 | 14.40 | 3.84 |
| Lu | 0.04 | 0.66 | 1.06 | 0.01 | 1.29 | 1.63 | 1.88 | 1.95 | 1.45 | 1.95 | 2.19 | 0.60 | 1.89 | 0.52 |
| Y | 5.28 | 131.00 | 73.50 | 0.54 | 88.60 | 118.00 | 131.00 | 137.00 | 102.00 | 136.00 | 146.00 | 118.00 | 141.00 | 115.00 |
| V | 21.10 | 73.70 | 149.00 | 1.48 | 8.22 | 13.60 | 8.14 | 7.94 | 10.10 | 12.30 | 22.10 | 15.40 | 26.40 | 11.50 |
| Cr | 5.00 | 12.10 | 23.50 | 1.20 | 5.78 | 8.08 | 6.04 | 6.28 | 6.00 | 5.65 | 8.78 | 9.65 | 27.90 | 8.10 |
| Co | 8.56 | 20.00 | 12.90 | 0.20 | 14.40 | 14.90 | 8.45 | 12.80 | 3.60 | 4.12 | 6.58 | 39.40 | 47.10 | 59.50 |
| Ni | 19.49 | 128.49 | 201.78 | 2.18 | 23.89 | 37.60 | 24.75 | 23.80 | 19.92 | 21.64 | 61.14 | 39.32 | 46.05 | 57.95 |
| Mo | 3.46 | 25.10 | 38.20 | 0.48 | 2.67 | 3.61 | 1.26 | 1.27 | 1.26 | 1.45 | 5.44 | 4.99 | 2.88 | 5.00 |
| Ag | 0.77 | 1.72 | 12.30 | 0.02 | 1.40 | 1.59 | 1.64 | 1.42 | 0.88 | 0.89 | 0.91 | 26.50 | 14.70 | 19.30 |
| As | 9.85 | 63.70 | 156.00 | 0.11 | 13.40 | 15.30 | 14.90 | 12.70 | 10.60 | 12.60 | 16.80 | 22.30 | 29.50 | 38.30 |
| Sb | 17.72 | 38.48 | 116.51 | 0.04 | 12.88 | 22.73 | 10.99 | 10.58 | 4.68 | 6.73 | 12.14 | 63.75 | 83.69 | 98.46 |
