# Supplementary material for: Studies on geochemical characteristics and biomineralization of Cambrian phosphorites, Zhijin, Guizhou Province, China
Source: PLoS One. 2023 Feb 10;18(2):e0281671. doi: 10.1371/journal.pone.0281671 (PMC9916593; doi:10.1371/journal.pone.0281671)
Supplement: S3 Table — (DOCX) [file pone.0281671.s003.docx]

**S3 Table** Calculated values of some geochemical parameters of the Zhijin phosphorites samples in the lower Cambrian strata.

| Sample | ZDMC-1-1 | ZDMC-2-1 | ZDMC-2-2 | ZDMC-3-1 | ZGZW-1-1 | ZGZW-1-2 | ZGZW-3-2 | ZGZW-3-3 | ZGZW-4-2 | ZGZW-5-1 | ZGZW-6-1 | ZGZW-6-3 | ZGZW-7-1 | ZGZW-8-2 |
| --- | --- | --- | --- | --- | --- | --- | --- | --- | --- | --- | --- | --- | --- | --- |
| ΣREE_La-Lu_ | 264.19 | 143.06 | 598.86 | 18.24 | 541.18 | 209.83 | 641.46 | 590.66 | 449.20 | 401.64 | 602.33 | 637.27 | 678.97 | 794.86 |
| ΣREY_La-Y_ | 374.19 | 205.66 | 705.86 | 24.48 | 637.68 | 288.13 | 756.46 | 678.26 | 615.20 | 547.64 | 681.83 | 725.27 | 788.97 | 942.86 |
| LREE_La-Nd_ | 209.60 | 112.12 | 408.60 | 14.05 | 398.20 | 171.40 | 471.00 | 461.40 | 353.70 | 314.80 | 463.90 | 492.10 | 495.20 | 539.30 |
| HREE_Er-Lu_ | 13.60 | 7.98 | 38.74 | 0.79 | 30.71 | 9.40 | 36.21 | 28.62 | 21.39 | 18.00 | 24.69 | 26.12 | 33.14 | 46.66 |
| MREE_Sm-Ho_ | 40.99 | 22.96 | 151.52 | 3.40 | 112.27 | 29.03 | 134.25 | 100.64 | 74.11 | 68.84 | 113.74 | 119.05 | 150.63 | 208.90 |
| (MREE/LREE)_N_ | 1.77 | 1.85 | 3.36 | 2.19 | 2.55 | 1.53 | 2.58 | 1.97 | 1.90 | 1.98 | 2.22 | 2.19 | 2.75 | 3.51 |
| (MREE/HREE)_N_ | 1.11 | 1.06 | 1.44 | 1.58 | 1.35 | 1.14 | 1.37 | 1.30 | 1.28 | 1.41 | 1.70 | 1.68 | 1.68 | 1.65 |
| Eu/Eu* | 0.94 | 1.08 | 1.12 | 3.83 | 1.04 | 1.07 | 1.05 | 1.00 | 1.08 | 1.13 | 1.52 | 1.42 | 1.41 | 1.35 |
| Ce/Ce* | 0.41 | 0.46 | 0.23 | 0.51 | 0.55 | 0.48 | 0.53 | 0.39 | 0.38 | 0.40 | 0.40 | 0.40 | 0.54 | 0.23 |
| Pr/Pr* | 1.28 | 1.24 | 1.57 | 1.17 | 1.37 | 1.27 | 1.36 | 1.32 | 1.35 | 1.30 | 1.29 | 1.29 | 1.30 | 1.51 |
| Y/Y* | 1.59 | 1.65 | 0.47 | 1.53 | 0.55 | 1.68 | 0.55 | 0.54 | 1.40 | 1.35 | 0.52 | 0.54 | 0.52 | 0.53 |
| (La/Sm)_N_ | 1.05 | 1.01 | 0.28 | 0.98 | 0.35 | 1.14 | 0.35 | 0.87 | 0.95 | 0.89 | 0.68 | 0.68 | 0.28 | 0.26 |
| (La/Yb)_N_ | 1.25 | 1.06 | 0.48 | 1.22 | 0.54 | 1.32 | 0.54 | 1.29 | 1.38 | 1.47 | 1.45 | 1.48 | 0.60 | 0.54 |
| (Dy/Sm)_N_ | 1.21 | 1.25 | 1.08 | 1.01 | 1.15 | 1.14 | 1.14 | 1.20 | 1.19 | 1.19 | 0.96 | 0.97 | 1.00 | 0.94 |
| La/Nd | 1.39 | 1.34 | 0.39 | 1.51 | 0.47 | 1.44 | 0.47 | 1.16 | 1.26 | 1.17 | 0.97 | 0.96 | 0.39 | 0.40 |
| Y/Ho | 40.59 | 42.30 | 12.74 | 38.76 | 14.67 | 43.74 | 14.43 | 14.15 | 36.97 | 35.52 | 13.83 | 14.36 | 14.01 | 14.23 |
| Er/Nd | 0.13 | 0.14 | 0.11 | 0.10 | 0.11 | 0.11 | 0.11 | 0.11 | 0.12 | 0.11 | 0.09 | 0.09 | 0.09 | 0.09 |
| V/Ni | 0.60 | 0.97 | 1.14 | 1.59 | 0.40 | 1.25 | 0.90 | 0.28 | 0.23 | 1.58 | 4.41 | 2.04 | 3.91 | 1.04 |
| V/Cr | 1.39 | 1.76 | 1.18 | 1.17 | 2.23 | 2.46 | 2.66 | 3.76 | 2.11 | 5.01 | 9.03 | 4.05 | 5.41 | 3.71 |
| Ni/Co | 0.88 | 1.25 | 1.54 | 5.74 | 5.09 | 9.51 | 4.42 | 14.02 | 8.62 | 2.22 | 2.55 | 2.49 | 2.88 | 3.77 |
|  |  |  |  |  |  |  |  |  |  |  |  |  |  |  |
| Sample | ZLX1-1 | ZLX1-3 | ZLX1-4 | ZLX2-1 | ZLX2-2 | ZLX2-5 | ZLX2-8 | ZLX3-1 | ZLX3-2 | ZGH-1 | ZGH-4-1 | ZGH-4-2 | ZGH-5-2 |  |
| ΣREE_La-Lu_ | 426.38 | 730.12 | 484.95 | 336.92 | 578.74 | 669.09 | 728.00 | 563.82 | 536.49 | 63.34 | 532.19 | 69.99 | 142.22 |  |
| ΣREY_La-Y_ | 585.38 | 882.12 | 570.75 | 473.92 | 677.24 | 802.09 | 879.00 | 675.82 | 627.89 | 86.84 | 595.09 | 93.59 | 159.32 |  |
| LREE_La-Nd_ | 337.70 | 538.60 | 378.40 | 267.90 | 452.20 | 491.10 | 500.90 | 410.60 | 409.20 | 51.54 | 406.70 | 49.29 | 127.16 |  |
| HREE_Er-Lu_ | 19.79 | 41.04 | 22.25 | 15.47 | 26.55 | 36.26 | 44.94 | 31.49 | 25.10 | 2.52 | 23.35 | 5.32 | 4.91 |  |
| MREE_Sm-Ho_ | 68.89 | 150.48 | 84.30 | 53.54 | 99.99 | 141.73 | 182.16 | 121.73 | 102.19 | 9.28 | 102.14 | 15.38 | 10.15 |  |
| (MREE/LREE)_N_ | 1.85 | 2.53 | 2.02 | 1.81 | 2.00 | 2.61 | 3.29 | 2.68 | 2.26 | 1.63 | 2.27 | 2.82 | 0.72 |  |
| (MREE/HREE)_N_ | 1.28 | 1.35 | 1.40 | 1.28 | 1.39 | 1.44 | 1.49 | 1.42 | 1.50 | 1.36 | 1.61 | 1.06 | 0.76 |  |
| Eu/Eu* | 1.06 | 1.03 | 1.12 | 1.05 | 0.96 | 1.28 | 1.07 | 1.16 | 1.50 | 1.14 | 1.03 | 3.04 | 0.92 |  |
| Ce/Ce* | 0.36 | 0.46 | 0.36 | 0.36 | 0.39 | 0.55 | 0.25 | 0.56 | 0.42 | 0.54 | 0.51 | 0.61 | 0.91 |  |
| Pr/Pr* | 1.34 | 1.36 | 1.35 | 1.36 | 1.33 | 1.30 | 1.53 | 1.29 | 1.28 | 1.18 | 1.29 | 1.15 | 1.10 |  |
| Y/Y* | 1.45 | 0.63 | 0.66 | 1.59 | 0.63 | 0.60 | 0.55 | 0.60 | 0.60 | 1.77 | 0.44 | 1.21 | 1.03 |  |
| (La/Sm)_N_ | 1.03 | 0.43 | 0.89 | 1.07 | 0.81 | 0.39 | 0.32 | 0.35 | 0.73 | 0.94 | 0.53 | 0.51 | 1.62 |  |
| (La/Yb)_N_ | 1.49 | 0.66 | 1.40 | 1.47 | 1.35 | 0.65 | 0.57 | 0.57 | 1.27 | 1.48 | 1.05 | 0.41 | 1.16 |  |
| (Dy/Sm)_N_ | 1.21 | 1.19 | 1.18 | 1.23 | 1.15 | 1.23 | 1.12 | 1.16 | 1.15 | 0.95 | 1.00 | 1.02 | 0.97 |  |
| La/Nd | 1.29 | 0.56 | 1.13 | 1.34 | 1.03 | 0.50 | 0.45 | 0.48 | 1.00 | 1.39 | 0.83 | 1.06 | 1.65 |  |
| Y/Ho | 38.04 | 16.65 | 17.13 | 41.90 | 16.53 | 15.57 | 14.52 | 15.95 | 15.65 | 46.53 | 12.07 | 34.45 | 25.71 |  |
| Er/Nd | 0.11 | 0.11 | 0.11 | 0.11 | 0.11 | 0.11 | 0.11 | 0.11 | 0.11 | 0.10 | 0.10 | 0.15 | 0.09 |  |
| V/Ni | 0.11 | 0.07 | 0.14 | 0.33 | 2.29 | 2.12 | 0.14 | 0.76 | 2.16 | 3.30 | 2.22 | 44.41 | 21.77 |  |
| V/Cr | 0.35 | 0.21 | 0.48 | 1.13 | 1.52 | 2.26 | 1.25 | 1.17 | 2.79 | 4.61 | 1.93 | 0.33 | 2.31 |  |
| Ni/Co | 13.95 | 31.28 | 4.98 | 4.91 | 3.83 | 2.92 | 11.90 | 1.28 | 0.56 | 1.06 | 3.56 | 0.03 | 0.72 |  |
|  |  |  |  |  |  |  |  |  |  |  |  |  |  |  |
| Sample | ZX-2 | ZX-4-1 | ZX-5 | ZX-7 | ZYCG-1 | ZYCG-2 | ZYCG-3 | ZYCG-4 | ZYCG-5 | ZYCG-6 | ZYCG-7 | ZYCG-8 | ZYCG-9 | ZYCG-10 |
| ΣREE_La-Lu_ | 12.74 | 345.25 | 488.53 | 1.82 | 510.30 | 588.99 | 763.80 | 779.29 | 568.88 | 746.56 | 703.01 | 294.44 | 784.65 | 312.53 |
| ΣREY_La-Y_ | 18.02 | 476.25 | 562.03 | 2.37 | 598.90 | 706.99 | 894.80 | 916.29 | 670.88 | 882.56 | 849.01 | 412.44 | 925.65 | 427.53 |
| LREE_La-Nd_ | 9.58 | 275.60 | 378.30 | 1.48 | 376.90 | 400.90 | 558.80 | 567.60 | 418.20 | 544.20 | 480.70 | 232.80 | 527.00 | 244.70 |
| HREE_Er-Lu_ | 0.59 | 14.63 | 23.15 | 0.09 | 29.30 | 39.15 | 44.06 | 45.01 | 33.68 | 44.71 | 48.69 | 12.92 | 46.86 | 12.62 |
| MREE_Sm-Ho_ | 2.57 | 55.03 | 87.08 | 0.25 | 104.10 | 148.94 | 160.94 | 166.68 | 117.00 | 157.65 | 173.62 | 48.72 | 210.79 | 55.20 |
| (MREE/LREE)_N_ | 2.43 | 1.81 | 2.08 | 1.52 | 2.50 | 3.36 | 2.61 | 2.66 | 2.53 | 2.62 | 3.27 | 1.89 | 3.62 | 2.04 |
| (MREE/HREE)_N_ | 1.61 | 1.39 | 1.39 | 1.04 | 1.31 | 1.40 | 1.35 | 1.36 | 1.28 | 1.30 | 1.31 | 1.39 | 1.66 | 1.61 |
| Eu/Eu* | 1.33 | 1.19 | 1.09 | 0.96 | 1.20 | 1.13 | 1.14 | 1.11 | 0.98 | 0.98 | 0.99 | 1.31 | 1.35 | 1.45 |
| Ce/Ce* | 0.55 | 0.48 | 0.44 | 0.88 | 0.58 | 0.21 | 0.53 | 0.53 | 0.51 | 0.47 | 0.19 | 0.43 | 0.24 | 0.44 |
| Pr/Pr* | 1.18 | 1.26 | 1.29 | 1.14 | 1.32 | 1.59 | 1.33 | 1.33 | 1.36 | 1.38 | 1.62 | 1.26 | 1.48 | 1.25 |
| Y/Y* | 1.58 | 1.55 | 0.55 | 1.65 | 0.54 | 0.52 | 0.52 | 0.52 | 0.52 | 0.52 | 0.51 | 1.61 | 0.48 | 1.46 |
| (La/Sm)_N_ | 0.50 | 0.96 | 0.82 | 0.71 | 0.34 | 0.30 | 0.32 | 0.32 | 0.35 | 0.36 | 0.35 | 0.86 | 0.24 | 0.77 |
| (La/Yb)_N_ | 1.00 | 1.55 | 1.28 | 0.90 | 0.50 | 0.50 | 0.50 | 0.51 | 0.52 | 0.53 | 0.53 | 1.49 | 0.51 | 1.54 |
| (Dy/Sm)_N_ | 0.87 | 1.14 | 1.16 | 0.82 | 1.16 | 1.07 | 1.14 | 1.18 | 1.21 | 1.24 | 1.21 | 1.06 | 0.98 | 1.07 |
| La/Nd | 0.89 | 1.32 | 1.20 | 1.21 | 0.44 | 0.42 | 0.42 | 0.42 | 0.45 | 0.45 | 0.46 | 1.18 | 0.36 | 1.05 |
| Y/Ho | 42.93 | 40.81 | 14.50 | 41.85 | 14.13 | 13.74 | 13.56 | 13.70 | 13.46 | 13.60 | 13.15 | 42.29 | 12.82 | 39.38 |
| Er/Nd | 0.10 | 0.11 | 0.12 | 0.13 | 0.11 | 0.11 | 0.11 | 0.11 | 0.11 | 0.11 | 0.11 | 0.11 | 0.10 | 0.10 |
| V/Ni | 1.08 | 0.57 | 0.74 | 0.68 | 0.34 | 0.36 | 0.33 | 0.33 | 0.51 | 0.57 | 0.36 | 0.39 | 0.57 | 0.20 |
| V/Cr | 4.22 | 6.09 | 6.34 | 1.23 | 1.42 | 1.68 | 1.35 | 1.26 | 1.68 | 2.18 | 2.52 | 1.60 | 0.95 | 1.42 |
| Ni/Co | 2.28 | 6.42 | 15.64 | 10.96 | 1.66 | 2.52 | 2.93 | 1.86 | 5.53 | 5.25 | 9.29 | 1.00 | 0.98 | 0.97 |
